# Generative artificial intelligence writing open notes: A mixed methods assessment of the functionality of GPT 3.5 and GPT 4.0

**DOI:** 10.1177/20552076241291384

**Published:** 2024-10-29

**Authors:** Anna Kharko, Brian McMillan, Josefin Hagström, Irene Muli, Gail Davidge, Maria Hägglund, Charlotte Blease

**Affiliations:** 1 Participatory eHealth and Health Data Research Group, Department of Women's and Children's Health, 8097 Uppsala University, Uppsala, Sweden; 2 Medtech Science & Innovation Centre, Uppsala University Hospital, Uppsala, Sweden; 3 School of Psychology, Faculty of Health, 6633 University of Plymouth, Plymouth, UK; 4 Centre for Primary Care and Health Services Research, The University of Manchester, Manchester, UK; 5 Digital Psychiatry, Department of Psychiatry, Beth Israel Deaconess Medical Center, Harvard Medical School, Boston, MA, USA

**Keywords:** Generative artificial intelligence, primary care, general practice, online record access, electronic health records, open notes, patient-centered care, documentation

## Abstract

**Background:**

Worldwide, patients are increasingly being offered access to their full online clinical records including the narrative reports written by clinicians (so-called “open notes”). Against these developments, there is growing interest in the use of generative artificial intelligence (AI) such as OpenAI's ChatGPT to co-assist clinicians with patient-facing documentation.

**Objective:**

This study aimed to explore the effectiveness of OpenAI's ChatGPT 3.5 and GPT 4.0 in generating three patient-facing clinical notes from fictional general practice narrative reports.

**Methods:**

On 1 October 2023 and 1 November 2023, we used ChatGPT 3.5 and 4.0 to generate notes for three validated fictional general practice notes, using a prompt in the style of a British primary care note for three commonly presented conditions: (1) type 2 diabetes, (2) major depressive disorder, and (3) a differential diagnosis for suspected bowel cancer. Outputs were analyzed for reading ease, sentiment analysis, empathy, and medical fidelity.

**Results:**

ChatGPT 3.5 and 4.0 wrote longer notes than the original, and embedded more second person pronouns, with ChatGPT 3.5 scoring higher on both. ChatGPT expanded abbreviations, but readability metrics showed that the notes required a higher reading proficiency, with ChatGPT 3.5 demanding the most advanced level. Across all notes, ChatGPT offered higher signatures of empathy across cognitive, compassion/sympathy, and prosocial cues. Medical fidelity ratings varied across all three cases with ChatGPT 4.0 rated superior.

**Conclusions:**

While ChatGPT improved sentiment and empathy metrics in the transformed notes, compared to the original they also required higher reading proficiency and omitted details impacting medical fidelity.

## Introduction

Worldwide, health institutions are increasingly opening online patient access to medical records via secure portals and apps.^1^ Online record access (ORA) can include test results, lists of medications, and narrative reports written by clinicians (the latter, often referred to as “open notes”).^2^ In some countries, the practice is advanced.^3,4^ In the United States from April 2021, the 21st Century Cures Act mandated that providers offer patients access to their online clinical records, without charge.^5^ In the Nordic countries, ORA has been implemented incrementally, starting around 2010.^1^ For example, in Finland, Omakanta, or My Kanta, was rolled out with implementation rolled out between 2010 and 2015.^6^ In Sweden, patients first obtained ORA through 1177 in 1 of 21 regions in 2012^7^ with countrywide implementation reached by 2018. In the United Kingdom, from November 2023 it became mandatory for general practitioners (GPs) working in NHS England to enable prospective ORA by default to patients aged 16 or older.^8^

In the era of open notes, the functionality of medical records is evolving. The record is no longer only an aide memoire or communication tool for clinicians but now has an additional purpose to rapidly convey health information to patients, and their caregivers.^1,9^ With the knowledge that patients may now be reading what they write, in some surveys physicians report changing how they document medical information, and the language they use.^10–12^ While the extent of changes to documentation post-ORA is not well understood,^13^ conceivably some modifications might be positive, for example removing dense medical terminology, problematic acronyms (e.g. “SOB” for shortness of breath, or “F/U” for follow up) or omitting potentially offensive medical vernacular (such as “*patient denies*,” or “*patient complains of*”). However, other changes might risk undermining the accuracy and completeness of records.^14,15^ Again, although few objective studies have explored the potential for additional work burdens of open notes,^16^ some clinicians report spending longer writing documentation for ORA.^12,17^ Some suggest that there is an essential tension between dual function documentation written for both clinicians and patients,^18^ and that notes should ideally be created for respective readerships.^18,19^

Increasingly, it is recognized that the use of generative artificial intelligence (AI) may offer a long-term strategy to assist clinicians with undertaking such documentation, including co-writing open notes.^19–21^ Increased accessibility of large language models (LLMs) such as OpenAI's Chat Generative Pre-trained Transformer (ChatGPT), Meta's Large Language Model Meta AI (LLaMA), and Google's Pathways Language Model 2 (PaLM2) make them particularly viable. These tools have the ability to recognize and summarize data,^22^ and to present content in a variety of requested styles including embedding empathic and supportive language.^23^ In addition, the speed of responses combined with their conversational fluency means uptake has been rapid.

Moreover, preliminary evidence already suggests that clinicians are adopting LLM-powered chatbots for a variety of tasks including assisting with documentation.^24,25^ In October 2023, a survey conducted with the American Psychiatric Association found that 44% of respondents had used ChatGPT 3.5 and 33% had used 4.0 “*to assist with answering clinical questions”* with 70% of psychiatrists believing that “*documentation will be/is more efficient”* as a result of these tools.^25^ Even more pressingly, conducted in February 2024, a study of 1006 UK GPs found that 20% reported using generative AI tools in clinical practice; of those who answered affirmatively and were invited to clarify further, 29% reported using these tools to generate documentation after patient appointments.^26^ These findings highlight the need for further research into the adequacy of these tools to assist with writing clinical notes.

Despite their considerable promise, these tools come with well-documented limitations.^27–29^ The nature of the datasets on which responses are trained is critical, and any biases embedded in the training set, or among human agents involved in labeling or training the AI, mean biases may become baked into responses. In addition, the more accessible LLM-powered chatbots are not exclusively trained on medical texts and treat the varied quality of information available on the internet indiscriminately. Furthermore, the routine under recruitment of female participants, racial and ethnic minorities, and seniors in research mean that disparities may already be embedded in published medical texts.^30–34^ Combined, these factors, influence the scope of coded and biased responses offered by LLM-chatbots.^35^

Other problems include lack of consistency in responses, and “hallucinations”—the tendency of LLM-chatbots to invent false information.^29^ Despite their fluency, these tools do not understand the information fed into them leading to a variety of elementary linguistic shortcomings, such as the inability to understand negation, and to be easily confused by word sentence order or rephrasing of questions.^27,36^ For example, inputting the same question to ChatGPT rarely elicits the same response.^37,38^ Yet, their quick and compelling conversational tone means LLM-chatbot responses can appear authoritative and factual, leading to risks of misinformation.^39,40^

Notwithstanding these shortcomings, LLM-powered chatbots carry enormous potential to co-assist clinicians when it comes to writing documentation. We emphasize that ChatGPT is not trained on medical data, specifically, and therefore other medical grade models such as Google's PALMMed2 may do a superior job writing documentation. However, given the commercial availability of ChatGPT, and the fact it appears to be the most widely adopted LLM chatbot,^26^ with preliminary studies indicating physicians are already using it, we have chosen to focus on GPT in this study.^25,26^ Furthermore, while the promise of documentation capacity frequently alluded to in academic medical journals,^41–43^ to date there is scarcely any experimental exploration of the effectiveness of LLM tools in writing open notes.^44^ In a randomized controlled study led by Baker et al.,^45^ ChatGPT generated longer and more detailed documentation compared with typing or dictation methods, however it also embedded errors and hallucinations.

To address current research gaps, we used ChatGPT^46^—the most widely adopted generative AI chatbot—to examine its ability to translate physician documentation into patient-facing notes. First, we aimed to compare the linguistic properties of the original and generated ChatGPT notes. Second, we aimed to assess the patient-centeredness of the original and ChatGPT notes by analyzing for readability and empathy. Third, we aimed to assess the medical fidelity of the generated notes.

## Methods

To answer the research questions, we carried out a study in the United Kingdom between October 2023 and January 2024, in which we used ChatGPT to transform fictional primary care notes into patient-facing notes.

### Materials

We used three fictional primary care notes written in the style of free text entries by GPs in England. These entries were devised by one of the authors (BM) who works as a GP in England. Each note was independently validated for authenticity by a panel of six UK-based GPs. Entries were devised to encompass three commonly presented chronic conditions in primary care: (a) a diagnosis of type 2 diabetes, (b) a diagnosis of major depressive disorder, and (c) a differential diagnosis where the probable opinion was bowel cancer (see Supplemental Appendix 1). Notes were devised to maximize authentic levels of detail including acronyms and potential for offensive language, and were deliberately fictionalized to avoid ethical concerns associated with using real patient clinical data, including potential for de-identification.^28,47^

### Procedure

Each fictionalized note was cut and pasted by author CB into ChatGPT together with following prompt, “*Write an understandable and empathic clinical note to be read by the patient described in this record:”*. We compared the responses of ChatGPT 3.5, which is free to users and is described by OpenAI as, “*Our fastest model, great for most everyday tasks”*, and ChatGPT 4.0, which functions behind a paywall, and is described as, “*Our most capable model, great for tasks involving creativity and advanced reasoning”.*^46^ This was done on 1 October 2023 and repeated on 1 November 2023 in light of findings that its outputs are inconsistent and may change over time.^48^ The resulting ChatGPT notes were then saved in a way preserving text formatting and layout (Supplemental Appendix 1).

## Data analysis

To measure the ***linguistic metrics*** of the original and generated notes, we assessed total word count, words per sentence (WPS), percentage of pronounce per note and percentage of abbreviations per note. To calculate these, AK used Linguistic Inquiry and Word Count software (LIWC-22, University of Texas at Austin).

To assess patient-centeredness, AK, JH, IM, and CB compiled readability and empathy metrics. ***Readability metrics*** included several metrics designed to assess the difficulty of reading a text by equating it to a projected reading grade level in the US school system. These were Flesch Reading Ease, Flesch-Kincaid Grade Level,^49^ and the Gunning Fog Index.^50^ We adopted the approach of using distinctive but complementary metrics to obtain a richer understanding of readability. For example, the Flesch Reading Ease and Flesch-Kincaid Grade Level use word syllable count to gauge readability while the Gunning Fox Index includes variables such as the proportion of complex words.

To derive ***empathy metrics***, we used both sentiment analysis and qualitative analysis. For the sentiment analysis, the original and ChatGPT notes were cleaned of stop words and punctuation, associating the data with a Word-Emotion Association lexicon,^51^ and calculating the percentages of emotion/sentiment words in each text. Eight emotions (anger, anticipation, disgust, fear, joy, sadness, surprise, and trust) and two sentiments (positive and negative) were considered. One word could be associated with several sentiments/emotions. JH conducted the analysis using R v4.2.2 (R Core Team).

The qualitative analysis employed a theoretically deductive thematic approach following the six-phase process outlined by Braun & Clarke.^52^ To promote theme saturation, all ChatGPT notes were analyzed together. Deductive qualitative analysis was chosen for the analysis of empathy because there is considerable latitude in how empathy is defined in medical contexts with the potential to lead to problematic inferences.^53,54^ Following previous work, we interpreted empathy as a multifaceted construct encompassing four dimensions: *affective empathy* (the capacity to feel what others are feeling which may include affective reactions), *cognitive empathy* (the capacity to identify, interpret and demonstrate understanding about another person's emotional state), *compassion/sympathy* (signals of warmth or feeling for someone's wellbeing), and *prosocial behavior* (signals of helping).^55–59^ While ChatGPT is incapable of feeling or grasping human experiences, perceptions of empathy may be embedded via textual cues. Using the four empathy dimensions, authors AK and CB marked phrases and sentences in the original and ChatGPT notes which signaled these dimensions. Once the text passages that signal empathy were extracted, AK coded them through low-level descriptive codes. Double-coding of passages was permissible and codes were not restricted to a single empathy dimension due to semantic overlap between dimensions. Once all text passages were coded, AK created a list of categories by merging semantically similar codes. The category list for each dimension was drafted by AK and finalized through iterative discussions between AK and CB.

To assess ***medical fidelity***, we convened a panel of UK-based GPs. In January 2024, author BM compiled a list of 10 GPs and contacted them individually with a document containing the original note for each case, as well as the ChatGPT notes with anonymized titles to hide the GPT version and time of collection. For each ChatGPT note, we asked the panel to judge whether they would or would not choose to use the note unchanged and to rate how well the generated note preserved the clinical accuracy of the original note (see Supplemental Appendices 1 and 2). A free text box was available to provide additional information. Qualitative analysis was carried out using inductive thematic approach following the six-phase process.^52^ Author AK coded the comments through low-level codes that were transformed into more abstract categories which gave rise to semantically connected themes. The final themes and categories were compiled through iterative discussions between AK and CB.

## Ethical considerations

Based on the NHS Research Ethics Committee Review Decision Tool, the study did not require ethical approval since we used only fictional clinical notes entered in a publicly accessible website and did not collect or analyze any personally identifying information but used only fictionalized material. The invited panel of experts were informed of the purposes of the investigation and provided written consent to the further analysis of the data and its use in educational and scientific publications.

## Results

### Linguistic metrics

Linguistic metrics of the notes are presented in Table 1. For all three cases, the ChatGPT 3.5 notes were longer than the original. The 4.0 notes were longer than the original but shorter than the 3.5 notes. The original note had no second person pronouns, for example, “you,” “your,” in the cases of diabetes and depression but some for cancer. Both ChatGPT 3.5 and 4.0 notes had a higher presence of second person pronouns across all cases with ChatGPT 3.5 being the highest.

**Table 1.** Linguistic metrics of original note and ChatGPT notes from 1 October 2023.

	**Original note**			**ChatGPT 3.5**			**ChatGPT 4.0**		
	Diabetes	Depression	Cancer	Diabetes	Depression	Cancer	Diabetes	Depression	Cancer
**Total words**, *n*	122	352	115	488	721	479	373	506	355
**WPS**, *m*	13.56	8.38	12.78	18.77	18.02	19.16	15.54	14.88	16.9
**Pronouns**, *%*									
1st person, sg.	0.82	0.28	0.87	3.07	1.94	1.67	1.34	–	2.25
1st person, pl.	–	–	–	2.87	2.22	2.71	2.95	–	1.13
2nd person	–	–	0.87	6.97	10.4	8.77	4.83	1.19	8.45
3rd person, sg.	–	0.28	0.87	–	–	0.21	–	7.71	0.28
3rd person, pl.	–	–	–	–	0.28	0.21	–	0.4	0.28
**Abbreviations**, *n*	17	4	10	3	1	5	11	1	8
Of which explained	–	–	–	1	–	5	8	–	7

Note: WPS: words per sentence. Pronouns detected by LIWC-22: 1st person, sg.—I, me, my, myself; 1st person, pl.—we, our, us, lets; 2nd person—you, your, u, yourself; 3rd person, sg.—he, she, her, his; 3rd person, pl.—they, their, them, themsel*. In the count of abbreviations, only unique instances were included and “XY” was excluded as it was placeholder initials for the fictitious note.

There were more abbreviations in the original notes than any of the generated ones. None of the abbreviations were explained in the original notes, ChatGPT 3.5 decoded most abbreviations, but ChatGPT 4.0 explained them more.

### Readability metrics

For all cases, the ease of readability decreased from the original note to ChatGPT 3.5 and 4.0 notes according to the Flesch Reading-Ease test, the Flesch-Kincaid Grade Level test, and the Gunning Fox Index (see Table 2). In almost all notes, the note generated by ChatGPT 3.5 required the highest reading proficiency.

**Table 2.** Readability metrics of original note and ChatGPT notes from 1 October 2023.

	**Original note**			**ChatGPT 3.5**			**ChatGPT 4.0**		
	Diabetes	Depression	Cancer	Diabetes	Depression	Cancer	Diabetes	Depression	Cancer
**Flesch Reading-Ease**									
Score	77.7	79.7	66.7	55.2	57.3	51.9	63	56.2	54.6
US grade level	7th	7th	8th–9th	10th–12th	10th–12th	10th–12th	8th–9th	10th–12th	10th–12th
**Flesch-Kincaid grade level**									
Score	4.9	4.2	6.5	10.1	9.8	10.8	7.8	9.2	9.9
**Gunning Fox Index**									
Score	7.1	5.9	6.5	13.3	12.5	10.8	9.2	9.2	9.9
US grade level	7th	6th	8th	College freshman	High-school senior	College sophomore	High-school freshman	High-school senior	High-school senior

Note: For the Flesch Reading-Ease test, the lower the score the more difficult the text is to read. For the Flesch-Kincaid Grade Level, the score represents a US grade level. For the Gunning Fox Index, the higher the score the more difficult the text is to read.

### Empathy metrics

**Figure 1.**
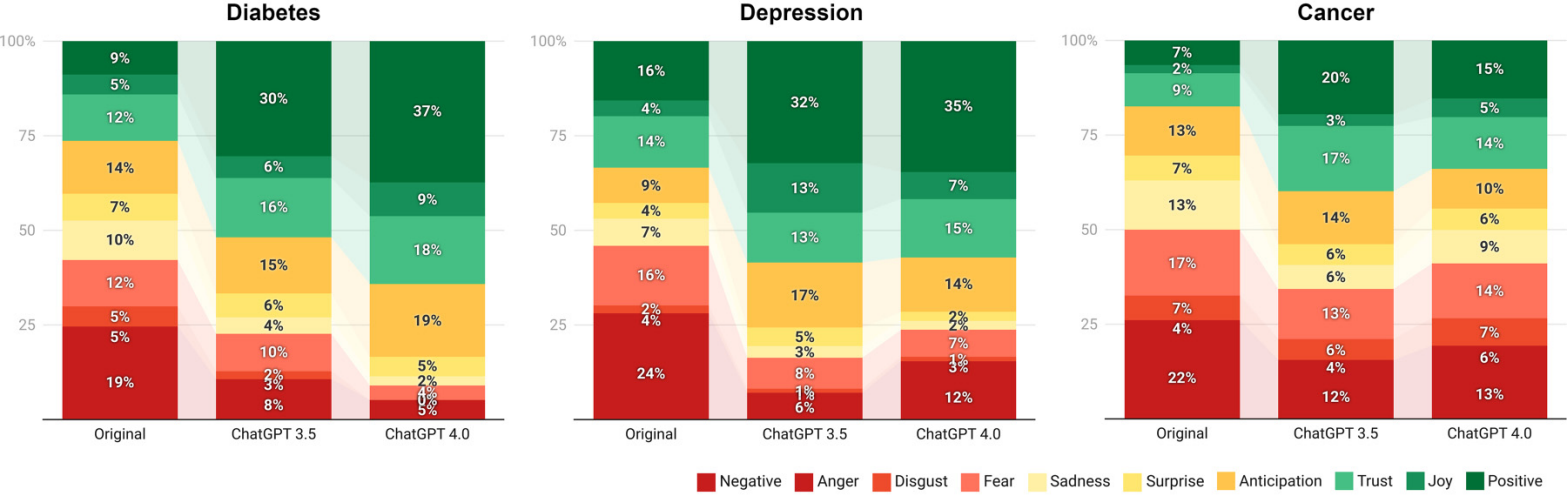
Frequency of identified emotions and sentiments in the original and ChatGPT notes from 1 October 2023.

Sentiment analysis of the original and ChatGPT notes showed a general increase in positive-leaning sentiment in the generated notes (see Figure 1).

Results from the qualitative analysis of the presence of the predefined empathy dimensions can be seen in Table 3. There were only four instances in the original notes that were coded as containing empathic text. In contrast, a variety of empathic text was identified in the ChatGPT notes.

**Table 3.** Dimensions of empathy in the original note and all ChatGPT notes broken into categories supplemented by exemplary quotes.

Dimension	**Original note**	**All ChatGPT notes**
**Affective**		
–	–	–
**Cognitive**		
Understanding context/stressors	–	*“Sally, it's evident that you've been through quite a lot in the past year.”*
Understanding upset state	*“Understandably upset.”*	*“I understand that this may come as a shock.”*
Acknowledgement of challenges	–	*“… it is clear that you have been facing some challenging times recently.”*
Reassurance	–	*“I want to reassure …”*
Understanding concerns	*“… understandably concerned.”*	*“I understand that the possibility of colorectal cancer can be concerning.”*
Diagnosis can be overwhelming	–	*“… managing a new diagnosis can feel overwhelming.”*
Journey metaphor	–	*“Please remember that managing diabetes is a journey, and it's completely normal to have questions and concerns along the way.”*
**Compassion/sympathy**		
Well wishes	–	*“I hope this message finds you well.”*
Offer of support	–	*“I've tasked a team member, XY, to assist you with the booking.”*
Appreciation	–	*“I appreciate the openness and honesty you brought to our conversation.”*
Positive outlook	–	*“… there is hope for a brighter future.”*
Not alone	–	*“Please remember that you don’t have to navigate this alone.”*
Patient is a priority	–	*“Your well-being is our top priority.”*
Praise	–	*“You have strengths and resilience within you.”*
Commitment to honesty	–	*“I want to be transparent and honest with you.”*
**Prosocial behavior**		
Referral to colleague/resources	–	*“I've sent you a link to the ‘Live Lighter’ program which offers guidance on this journey.”*
Explanation	–	*“I wanted to take a moment to discuss the findings from your recent appointment …”*
Encouragement to reach out	–	*“Please don't hesitate to reach out if you have any questions or concerns in the meantime.”*
Offer of support	*“Advised support available …”*	*“…we are here to support you every step of the way.”*
Next steps in care	*“I will call with results.”*	*“We have scheduled a follow-up appointment in two weeks.”*
Commitment to help	–	*“… we are committed to providing you with the best care possible.”*
Not alone	–	*“Please remember that you don't have to go through this alone, and there are people who care about your well-being, including your mum and brother.”*
Journey metaphor	–	*“Together, with the right support and resources, we will navigate this journey.”*
Promise of personal involvement	–	*“I will personally call you as soon as the results come in to discuss them with you.”*

Note: Text passages could signal more than one empathy dimension and category, which lead to same categories appearing in multiple dimensions.

### Medical fidelity

Six out of the 10 invited GPs judged the medical fidelity of the ChatGPT notes (see Table 4).

**Table 4.** Medical fidelity measures of original note and ChatGPT notes from 1 October 2023.

	**Original note**			**ChatGPT 3.5**			**ChatGPT 4.0**		
	Diabetes	Depression	Cancer	Diabetes	Depression	Cancer	Diabetes	Depression	Cancer
**Would you use the note generated by ChatGPT unchanged as it is?** *n (%)*									
Yes	–	–	–	0	2 (66.6)	5 (100)	3 (50)	6 (100)	6 (100)
No	–	–	–	6 (100)	4 (33.3)	0	3 (50)	0	0
**How well does ChatGPT preserve the clinical detail of the original note?** *median*									
Rating	–	–	–	5	6	7	7	6	6

Note: The Likert scale to rate the preservation of clinical detail ranged from “1 – Not at all” to “7 – Fully preserves clinical detail.” Calculations were made excluding missing data.

Seventy free-text comments were left by the panel of GPs giving additional insight into their opinion on the generated notes. Emerging themes and categories are illustrated in Table 5.

**Table 5.** Themes and categories emerging in the free-text comments left by GPs about the ChatGPT notes.

Theme and categories	Example quote
**Positive opinions**	
Liked the content	*“Great letter, with everything required.” [GP3]* *“Safe translation of requirements.” [GP5]*
Liked the tone/language	*“… empathetic without being scary or minimizing the potential diagnosis.” [GP2]*
Liked the layout/structure	*“looks very professional similar to a structured hospital discharge summary.” [GP1]*
Could use with changes	*“I think I could use this one. The only additional bit of detail I would include would be my impression or diagnosis.” [GP1]*
Patient-centered	*“… it did a fairly good job of picking out the bits that the patient was interested in.” [GP4]*
**Negative opinions**	
Did not like the content	*“I would not want to add addition phrases such as do not hesitate to reach out to us (as this will create unrealistic expectations for English primary care context).” [GP3]*
Did not like the tone/language	*“I’d be curious about what patients think of it and if it's too overfriendly/patronizing.” [GP2]*
Did not like the layout/structure	*“Starting the letter off by saying “clinical update” implies this is new information rather than a recap of the appointment.” [GP1]*
Would not use	*“Would definitely not use this text. Far too much jargon. What is a ‘positive finding’ – could mean something bad or good!” [GP5]*
Not patient-centered	*“Far too much info here and could be very confusing for a lay person.” [GP5]*
Missing content	*“It ignored the whole of the examination which feels more like it didn’t know what to do with it rather than a deliberate choice.” [GP4]*
Too long/verbose	*“Again, it is VERY long and this would be a barrier to usability by the doctor in the real world, if this is the only version recorded, as wouldn’t be able to scan it quickly.” [GP2]*
Unnecessary information	*“Not sure that the apology for booking the patient with the incorrect clinician is needed.” [GP6]*
Hallucination	*“It's intriguing to see all the letters ‘strongly recommend’ weight loss when this isn’t even implying in the original notes.” [GP4]*

## Discussion

### Main findings

The study aimed to assess the effectiveness of using LLM-chatbots in writing patient-facing clinical documentation. We asked ChatGPT to rewrite three fictitious clinical notes in an understandable and empathic manner for the patient. The generated notes were longer and required higher reading proficiency but contained more positive sentiment and signaled several dimensions of empathy. The medical fidelity of the original notes appeared high but was not always preserved.

All ChatGPT notes increased in total length as well as sentence length compared to the original. Therefore, it is unsurprising that the readability tests indicated a need for a higher reading proficiency, as readability formulas are based on sentence and word lengths.^49^ Notably, the original notes comprised multiple medical abbreviations, none of which were decoded or explained, in keeping with traditional clinical documentation practices. In contrast, ChatGPT notes reduced the use of abbreviations and usually explained most of them. Arguably this approach enhances readability as previous research has shown that patients find the use of medical abbreviations and jargon confusing when reading their notes.^60,61^

Empathy-signaling language was almost completely absent in the original notes, which was in stark contrast to the generated notes. The generated notes implied understanding of the hypothetical patient's emotional state, provided reassurance and validation, exhibiting cognitive empathy. Compassion and sympathy were also projected by ChatGPT, for example, through offers of support and well wishes, as well as through commitments to prioritizing the patient and not leaving them alone. Empathy was also expressed through descriptions of various prosocial behavior such as explanations, encouragement to contact the note writer, or further referrals. Affective empathy, a dimension of empathy associated with affective responses and “catching” another individual's feelings, was unsurprisingly lacking in both the original and generated notes.

While rewriting the notes in an understandable and empathic language was explicitly requested in the prompt, we also assessed whether ChatGPT maintained the medical fidelity of the original notes as it is reasonably expected of a clinical note. Medical fidelity ratings varied as well as seen in the number of GPs who reported they would use the generated notes unchanged. Analysis of the free-text comments revealed potential reasons for that. GPs liked various aspects of the generated notes. They appreciated the content, the layout of the note as well as the tone, and found they could envision using the note with some alterations to the content. However, sometimes GPs did not appreciate the structure or the content, finding crucial details to be missing or incorrect assumptions to have been made extrapolating beyond the original note. Such hallucinations could pose potential ethical and legal risks, as noted in some comments (*“My main concern about the ChatGPT note is that it does not include the examination findings which is a medicolegal issue.” [GP2]*).

While GPs found the tone of some of the notes acceptable, at other times they wondered if the documentation appeared overly friendly or was suitable in a British context. Conceivably, some patients may have been offended but others may have found the responses beneficial. The tone and language that ChatGPT uses, and cultural acceptability to different readers is receiving increasing attention. As previously explained, ChatGPT has been commonly perceived as authoritative due to the adoption of a confident tone.^29^ In our study, guided by the prompt for an empathic note, ChatGPT adopted a friendly tone uncharacteristic for the typical British context. This is likely due to the overrepresentation of US-originating text in the training of GPT, though what training materials were used has not been divulged by OpenAI.^62^ While this may not pose a problem in the lay use of ChatGPT, it may hinder its adoption in cultural and clinical settings outside the United States or require additional editing by physicians who use these tools.

### Comparison with previous work

Our study supports previous research by Baker et al.^45^ which shows ChatGPT writes longer and more detailed notes. As reported in that study, our study suggests that ChatGPT has the potential to improve clinical documentation by producing more comprehensive and organized notes. While our study found that fidelity was preserved for some notes, like the study by Baker, our panel of doctors also detected exclusions of information, and potential hallucinations.

Applying readability metrics to analyze note content, in line with Pradhan et al.^63^ who investigated the use of generative AI to write educational materials for cirrhosis, we found that ChatGPT, and especially version 3.5, responses were more demanding requiring higher reading grades than the original GP note. However, as noted earlier, some aspects of the documentation such as unpacking abbreviations may have mitigated this, and further research is needed to gauge patients’ opinions. Indeed, should patients use the internet to supplement their understanding about clinical documentation, as surmized by Blease,^20^ a study by Walker et al.^64^ reported that version 4.0 embeds comparable quality of information to static internet searches.

Ayers et al.^23^ reported that ChatGPT offered more empathic responses than clinicians, and our study supports this finding. Advancing this work, we probed the generated responses to analyze different dimensions of empathy and found ChatGPT offered a variety of signatures of empathy. An experimental study into lay perspectives on physician empathy by Gerger et al.^54^ found that compared with nonempathic interactions, quality-of-care was rated higher when physicians reacted with cognitively empathic or compassionate responses; with no significant difference reported between affective empathy and no empathy which were rated as offering lower quality care. Although our study did not include patient perspectives, given that generated responses were particularly high on cognitive empathy, compassion/sympathy, and prosocial behavior, our findings may suggest that patients might consider its documentation to be empathic. This is something that deserves further scrutiny, including whether patients can discern the difference between notes written by GPs and those written with the assistance of AI.

Notably, other research has examined the ethical perspectives on generative AI assisting with documentation, weighing up the benefits and risks especially with respect to exposing patients’ private and sensitive health information to third parties via these tools.^19,20,47^ Allen et al.^65^ argue that, in light of the quality of disclosures that clinicians currently offer to patients, there may be an important role for generative AI in augmenting and strengthening patient autonomy by improving informed consent processes. The present empirical study does not directly resolve such concerns but can help inform answers to those questions by offering further information on the quality of documentation that ChatGPT can offer.

Regulation of generative AI is evolving rapidly.^66,67^ In the European context, including England, ORA is widespread but authorities, including in the EU, are currently reviewing whether, without obtaining informed consent, OpenAI's ChatGPT complies with General Data Protection Regulations and meets the requirement that individual patient consent is not required for public health justifications.^68^ Already in the United States such tools will imminently become embedded in electronic health systems to assist with administrative tasks. For example, Epic, the US software vendor with the largest national share of hospital electronic medical records^69^ is piloting the integration of ChatGPT services^70^ aimed at compliance with the Health Insurance Portability and Accountability Act (HIPAA) which lays out federal standards for protecting patients’ sensitive health information from being shared by “covered entities”—that is providers—to other third parties. Furthermore, an Azure HIPAA compliant ChatGPT 4.0 service already exists^71^ with new speech-to-text innovations underway in this space.^72^ Despite regulatory and implementation advances, it is unclear how legislation will intersect in a practical way with these tools in clinical practice.^20^ Medical bodies have issued advice about the ethical use of these tools, although guidance has been criticized as limited.^73–77^ Despite the lack of guidance physicians already report using generative AI to assist with their job.^24,25^

### Strengths and limitations

This study has some strengths and limitations. The inclusion of diverse cases within primary care and fictional notes written by a practicing UK GP and authenticated by a panel of practicing GPs are strengths of the study. Our study compared the quality of the original note with both ChatGPT 3.5 and 4.0 responses, a consideration that is important if we are to compare these tools with current practice. Going beyond other studies, we also examined empathy as a multidimensional construct, to explore the signature of empathic cues in more detail in the documentation than in other studies.^23,78^ Another strength was employing linguistic metrics to assess pronoun usage, and an original finding of the paper was higher adoption of more directive, second-person pronouns, in ChatGPT writing patient-facing notes.

The study has several limitations. The cases were restricted to three fictional clinical notes to uphold privacy and confidentiality; conceivably, however, use of a wider range of documentation written by actual doctors could have influenced our findings. The prompt was also limited to a single sentence, without the provision of examples or followed by corrective feedback. While this was done in an attempt to recreate a typical ChatGPT user, prompt engineering in a healthcare context is a rapidly developing.^79^ In addition, our nonpurposive sample of GPs rating the documentation was small and may have affected the findings. Another limitation was that we used generative AI to rewrite documentation without input from GPs. However, it is not envisaged that these tools are close to replacing or disintermediating doctors in writing documentation.^22^ Rather, commentators propose that if these tools comply with patient privacy and are adopted in the future, clinicians may always be required to act as overseers to ensure note accuracy, appropriateness, and safe use.^19,29^ A potential limitation of sentiment analysis is that some words could be considered questionable without additional contextual information, for example the word “including” might be viewed as a positive sentiment within the phrase “including blood in your stool.” The use of a deductive qualitative approach to empathy analysis helped to supplement this limitation. Our study also did not include patients’ perspectives on the content, style, and readability of ChatGPT outputs; ultimately, however, patients’ views will be critical to understand the adequacy of these tools in assisting with documentation, including how doctors should edit generative AI notes.

Further experimental studies are required to investigate the adequacy of these tools in assisting with clinical documentation, including how they respond to different prompts.^38^ For example, studies could examine repeated prompts of the same fictional case to assess long-term medical fidelity and test the temporal consistency of content changes. Further, a variety of generative AI tools trained on clinical data are increasingly being adopted in practice. Studies should also examine physicians views about the ease of use of these tools within clinical workflow, and their potential efficiency to assist with documentation.^80,81^ Finally, more research is needed to explore patients’ opinions on clinical documentation created, or co-created by generative AI. For example, studies could explore how patients perceive “empathy” that is generated by chatbots.

## Conclusions

Compared with fictionalized GP notes across three case studies, ChatGPT 3.5 and 4.0 wrote longer notes, embedding higher presence of second person pronouns. ChatGPT also decoded medical abbreviations, but readability metrics showed that the generated notes required a higher reading proficiency, with ChatGPT 3.5 demanding the most advanced level. Across all notes, ChatGPT offered higher signatures of empathy across cognitive, compassion/sympathy, and prosocial cues. Medical fidelity ratings varied across all three cases with version 4.0 rated superior. Despite this, some GPs reported that they would be willing to use the generated notes unchanged. Our study highlights the need for deeper, ongoing analysis of the quality of clinical documentation generated by LLM-powered chatbots. Finally, our study highlights the need for greater guidance among clinicians about how to adopt these tools safely and ethically.

## Supplemental Material

sj-docx-1-dhj-10.1177_20552076241291384 - Supplemental material for Generative artificial intelligence writing open notes: A mixed methods assessment of the functionality of GPT 3.5 and GPT 4.0 Supplemental material, sj-docx-1-dhj-10.1177_20552076241291384 for Generative artificial intelligence writing open notes: A mixed methods assessment of the functionality of GPT 3.5 and GPT 4.0 by Anna Kharko, Brian McMillan, Josefin Hagström, Irene Muli, Gail Davidge, Maria Hägglund and Charlotte Blease in DIGITAL HEALTH

sj-docx-2-dhj-10.1177_20552076241291384 - Supplemental material for Generative artificial intelligence writing open notes: A mixed methods assessment of the functionality of GPT 3.5 and GPT 4.0 Supplemental material, sj-docx-2-dhj-10.1177_20552076241291384 for Generative artificial intelligence writing open notes: A mixed methods assessment of the functionality of GPT 3.5 and GPT 4.0 by Anna Kharko, Brian McMillan, Josefin Hagström, Irene Muli, Gail Davidge, Maria Hägglund and Charlotte Blease in DIGITAL HEALTH
