# Supplementary material for: Generative artificial intelligence writing open notes: A mixed methods assessment of the functionality of GPT 3.5 and GPT 4.0
Source: Digit Health. 2024 Oct 29;10:20552076241291384. doi: 10.1177/20552076241291384 (PMC11528788; doi:10.1177/20552076241291384)
Supplement: sj-docx-1-dhj-10.1177_20552076241291384 - Supplemental material for Generative artificial intelligence writing open notes: A mixed methods assessment of the functionality of GPT 3.5 and GPT 4.0 [file sj-docx-1-dhj-10.1177_20552076241291384.docx]

**Case 1: Diabetes**

Original Note

|  |
| --- |

History:
Asked tci to discuss HbA1c (63.3). Should have been booked in with RT but reception have booked in with me. Bloods from annual htn review. Already on a statin. Reports PUing more frequently for last few months and feeling more tired & thirsty.
Examination:
HR 94, BP 123/69, R 16, T36.6, Sats 98, weight 99.8kg (BMI 33.5)
Diagnosis:
Type II diabetes mellitus
Intervention Plan:
Explained diagnosis of T2DM. Understandably upset. Quick chat about low GI foods.
Advised support available – keen on f2f DESMOND – task to XY to book on
Start Metformin - 500 mg od for 1 week, then 500 mg bd for 1 week
Weight loss advised – sent link to ‘Live Lighter’
Booked in with XY in 2w time

|  |
| --- |

**Case 1: Diabetes**

ChatGPT 3.5 Note Oct 1^st^, 2023

|  |
| --- |

Clinical Note for Patient with Type II Diabetes Mellitus:

Dear [Patient's Name],

I hope this message finds you well. I wanted to provide you with a summary of our recent appointment and outline the plan we discussed to manage your health.

During our consultation, we discussed your HbA1c level, which was measured at 63.3. This raised some concerns, and it appears there was a booking error that led you to see me instead of the diabetes team. I understand that this might have been unexpected and stressful for you, and I want you to know that I am here to support you through this journey.

We also discussed your annual hypertension review, where we noticed that you have been urinating more frequently and experiencing increased fatigue and thirst over the last few months. These symptoms are indicative of Type II diabetes mellitus, which we have now diagnosed. I understand that receiving this diagnosis can be quite overwhelming, and I want you to know that it's okay to feel upset or worried.

During our appointment, we briefly touched on the importance of incorporating low glycemic index (GI) foods into your diet. This can be an essential part of managing your diabetes effectively. Additionally, I mentioned that there are support programs available to help you better understand and manage your condition. You expressed interest in the face-to-face DESMOND program, which is an excellent choice. I have tasked XY to assist you in booking your spot in this program, which I believe will provide you with valuable information and guidance.

To help manage your diabetes, we have initiated treatment with Metformin. You will start with 500 mg once daily for the first week and then increase to 500 mg twice daily in the following week. It's important to follow this medication schedule as prescribed, and we will closely monitor its effects during your future appointments.

In addition to medication, I strongly advise focusing on weight loss as part of your diabetes management plan. I have sent you a link to the 'Live Lighter' resource, which provides valuable information on adopting a healthier lifestyle, including dietary changes and exercise. This will complement the medication and help improve your overall well-being.

Lastly, I have scheduled your next appointment with XY in two weeks' time to review your progress, address any concerns, and make any necessary adjustments to your treatment plan.

Please remember that managing diabetes is a journey, and it's completely normal to have questions and concerns along the way. I am here to support you every step of the way, and together, we will work towards better health and well-being.

If you ever need to discuss anything further or have questions, please do not hesitate to reach out. Your health is our priority, and we are committed to helping you achieve the best possible outcomes.

Take care and stay positive.

Warm regards,

[Your Name]

[Your Title]

[Medical Practice/Institution Name]

[Contact Information]

|  |
| --- |

**Case 1: Diabetes**

ChatGPT 4.0 Note Oct 1^st^, 2023

|  |
| --- |

**Clinical Note For Patient**

**Date**: [Insert Date]

**Patient**: [Patient's Full Name]

**Visit Reason**: Discussion of recent blood test results and reported symptoms.

**History**:

Dear [Patient's Name],

Today, we discussed your recent blood test results which showed an HbA1c level of 63.3. Originally, you were to be seen by RT, but there seems to have been a mix-up in scheduling. We also talked about the results of your annual hypertension review and the fact that you're currently on a statin. You mentioned experiencing increased frequency of passing urine over the past few months and feelings of increased fatigue and thirst.

**Physical Examination**:

Heart Rate (HR): 94 beats per minute

Blood Pressure (BP): 123/69 mmHg

Respiratory Rate (R): 16 breaths per minute

Temperature (T): 36.6°C

Oxygen Saturation (Sats): 98%

Weight: 99.8kg

Body Mass Index (BMI): 33.5

**Diagnosis**:

Type II Diabetes Mellitus (T2DM).

**Intervention Plan**:

I explained the diagnosis of T2DM. It's natural to feel a range of emotions upon hearing such news, and I noticed you were understandably upset. We briefly discussed the benefits of incorporating low glycemic index (GI) foods into your diet.

Here's our plan moving forward:

1. **Education**: We talked about the support that's available to you. You expressed interest in a face-to-face DESMOND session. I've tasked a team member, XY, to assist you with the booking.
2. **Medication**: We'll start with Metformin. For the first week, take 500 mg once daily. After that, for the next week, take 500 mg twice daily. We'll review how you're feeling and make adjustments if necessary.
3. **Lifestyle Changes**: Given your weight and BMI, we discussed the benefits of weight loss, especially in managing T2DM. I've sent you a link to the 'Live Lighter' program which offers guidance on this journey.
4. **Follow-up**: I've scheduled another appointment for you with XY in 2 weeks to see how things are going and make sure we're on the right track.

**Final Thoughts**:

[Patient's Name], managing a new diagnosis can feel overwhelming. Remember, you're not alone in this. Together, with the right support and resources, we will navigate this journey. If you have any questions or concerns before our next meeting, please don't hesitate to reach out.

Take care,

[Your Name]

[Your Credentials]

|  |
| --- |

**Case 1: Diabetes**

ChatGPT 3.5 Note Nov 1^st^, 2023

|  |
| --- |

Clinical Note for Patient:

Patient Name: [Patient's Name] Date of Visit: [Date of Visit] Medical Record Number: [Patient's MRN]

Dear [Patient's Name],

I hope this note finds you well. I wanted to take a moment to discuss the findings from your recent appointment and provide you with some information about your health.

During our examination, we reviewed your blood test results, which showed an HbA1c level of 63.3. This is an indicator of your blood sugar control over the past few months, and it is higher than what we would like to see. Additionally, you mentioned that you have been urinating more frequently, feeling more tired, and experiencing increased thirst. These symptoms are important signs that we need to address.

Based on your medical history and the results of our examination, I have diagnosed you with Type II diabetes mellitus. I understand that this may come as a shock, and I want you to know that you are not alone in this journey. Diabetes is a manageable condition, and there are steps we can take to help you live a healthy and fulfilling life.

During our appointment, we briefly discussed the importance of incorporating low glycemic index (GI) foods into your diet. These foods can help stabilize your blood sugar levels. I have also informed you about a program called "DESMOND," which provides face-to-face support and education for individuals with diabetes. I believe it could be very beneficial for you, and I have tasked our team to help you schedule an appointment for this.

In addition to dietary changes and educational support, I am prescribing Metformin, a medication commonly used to manage blood sugar levels. You will start with a dose of 500 mg once daily for the first week and then increase to 500 mg twice daily after that. This medication, combined with lifestyle modifications, will help us work towards better blood sugar control.

Furthermore, I strongly recommend focusing on weight loss as it can significantly improve your diabetes management. I have provided you with a link to 'Live Lighter,' a resource that can assist you in making healthier choices and achieving your weight loss goals.

To ensure that we closely monitor your progress, I have scheduled a follow-up appointment with our colleague XY in two weeks. During this visit, we will assess how you are responding to treatment and make any necessary adjustments to your plan.

I understand that receiving a diabetes diagnosis can be overwhelming, but please know that our team is here to support you every step of the way. If you have any questions or concerns between now and your next appointment, do not hesitate to reach out to us. Your health and well-being are our top priorities.

Please take good care of yourself and follow the recommended steps to manage your diabetes effectively. With dedication and the right support, you can lead a healthy and fulfilling life despite this diagnosis.

Sincerely,

[Your Name] [Your Title] [Medical Practice Name] [Contact Information]

|  |
| --- |

**Case 1: Diabetes**

ChatGPT 4.0 Note Nov 1^st^, 2023

|  |
| --- |

Subject: Follow-Up and Care Plan for Your Recent Appointment

Dear [Patient's Name],

I hope this message finds you in good health and spirits. I wanted to follow up on our recent appointment to ensure that you have all the information and support you need. During our discussion, we talked about your blood test results, particularly your HbA1c level, which was 63.3. This level is higher than normal, leading us to diagnose Type 2 Diabetes Mellitus (T2DM). I understand that this news might have been unexpected and possibly overwhelming.

I want to assure you that you are not alone in this journey. Diabetes is a common condition, and with the right care and lifestyle changes, many people lead full, healthy lives. We have already started you on Metformin, a medication to help manage your blood sugar levels. Please remember to start with 500 mg once daily for the first week, and then increase to 500 mg twice daily in the second week.

I noticed that you have been experiencing increased thirst, tiredness, and more frequent urination over the last few months. These are common symptoms of diabetes, and with the intervention plan we have in place, we aim to alleviate these symptoms.

I encourage you to look at the 'Live Lighter' resource I sent you, as it provides helpful information on leading a healthier lifestyle, which is crucial in managing diabetes. We discussed the importance of low Glycemic Index (GI) foods, and incorporating more of these into your diet can help in managing your blood sugar levels.

I understand that managing a new diagnosis can be challenging, and I want to emphasize that support is available. I have referred you to the DESMOND program for face-to-face support and education on managing diabetes. My colleague, XY, will be in touch to book your session.

Lastly, I have scheduled a follow-up appointment for you with XY in two weeks' time. This will be an opportunity to discuss how you are managing and make any necessary adjustments to your care plan. In the meantime, if you have any questions or concerns, please do not hesitate to reach out.

Please remember, you are not alone in this, and we are here to support you every step of the way.

Kind regards,

[Your Name] [Your Title and Contact Information]

|  |
| --- |

**Case 2: Cancer**

Original Note

|  |
| --- |

Asked tci as iron deficiency anaemia. blood mixed with stool several occasions over last few weeks. See photo in record attachments. Not weighed self but gone down a notch on belt buckle and clothes feel looser. No abdo pain. Bowels looser recently. Father died of cancer in his 60’s but wasn;t talked about so not sure what type.

Examination:

abdo SNT no masses, PR no masses, no blood on glove, weight 82kg

Diagnosis:

?colorectal Ca, ?IBD

Intervention Plan:

urgent FIT – given pot needs U&Es, will go to drive through this afternoon, request on ICE await FIT, will likely need 2ww lower GI discussed possibility of Ca, understandably concerned. I will call with results.

|  |
| --- |

**Case 2: Cancer**

ChatGPT 3.5 Note Oct 1^st^, 2023

|  |
| --- |

Dear [Patient's Name],

I hope this message finds you well. I wanted to provide you with a clinical update regarding your recent visit to our clinic. During our consultation, you mentioned experiencing some concerning symptoms, including blood in your stool over the past few weeks, looser bowel movements, and an unexplained decrease in your weight. I understand that this has caused you significant worry and uncertainty.

Upon examining you, I did not find any masses or abnormalities in your abdomen or rectum. Additionally, there was no sign of blood during the rectal examination, which is a positive observation. Your current weight is 82 kilograms, and it's important to note that you've noticed a change in your body, with your clothes feeling looser and your belt buckle moving down a notch. I appreciate your openness in sharing your family history, specifically about your father's passing from cancer in his 60s, although you're unsure about the exact type of cancer.

Given your symptoms and concerns, I am recommending several steps to further evaluate your condition:

1. Urgent FIT (Fecal Immunochemical Test): You've been provided with a FIT kit, which you can use at home to collect a stool sample. This test will help us determine if there is any hidden blood in your stool, which could be indicative of various conditions, including colorectal cancer or inflammatory bowel disease (IBD). Please follow the instructions provided with the kit and return it as soon as possible.
2. Blood Tests (U&Es): To gather more information about your overall health, we will need to perform blood tests. You can visit the drive-through facility this afternoon for this purpose. We have placed a request for these tests in your electronic medical record (ICE).
3. Await FIT Results: Once we receive the results of the FIT, we will review them thoroughly. Depending on the findings, it may be necessary for you to have a "Two-Week Wait" (2ww) referral for a lower gastrointestinal examination. This is a precautionary step to ensure we thoroughly investigate any potential issues.

I understand that the possibility of colorectal cancer is understandably concerning to you. Please know that we are committed to providing you with the best care and support throughout this process. I will personally reach out to you as soon as we have the results of your FIT test to discuss the next steps and answer any questions you may have.

Your health and well-being are our top priorities, and we are here to support you every step of the way. Please don't hesitate to reach out if you have any immediate concerns or if you need further clarification about your condition and the upcoming tests.

Wishing you strength and courage during this challenging time. We will work together to determine the best course of action and ensure your health is well looked after.

Sincerely,

[Your Name]

[Your Title]

[Clinic Name]

|  |
| --- |

**Case 2: Cancer**

ChatGPT 4.0 Note Oct 1^st^, 2023

|  |
| --- |

**Clinical Note for [Patient's Name]**

**Date**: [Today's Date]

**Visit Summary:**

Dear [Patient's Name],

Thank you for coming in today and for sharing your concerns with me, especially considering your family's history. It's absolutely valid to be proactive about our health, especially when certain symptoms arise.

**Primary Concerns:**

You've noticed blood mixed with your stool on several occasions over the last few weeks.

Even though you haven't weighed yourself, you've mentioned that you've gone down a notch on your belt buckle and your clothes feel looser.

Your bowel movements have become looser recently.

No abdominal pain reported.

Your father unfortunately passed away from cancer in his 60s, though the specific type was not discussed in your family.

**Examination Findings:**

Your abdomen was soft and non-tender (SNT) with no palpable masses.

During the rectal examination, I didn't feel any masses and there was no blood evident on my glove.

Your current weight is recorded at 82kg.

**Possible Diagnoses:**

Colorectal Cancer (Ca)

Inflammatory Bowel Disease (IBD)

**Next steps:**

I've given you a pot for an urgent Faecal Immunochemical Test (FIT) to check for blood in your stools that might not be visible to the naked eye.

You'll need some blood tests (U&Es). Fortunately, you can get these done at the drive-through later today; the request has already been placed on the ICE system.

Once we receive the results from the FIT, you may need a more detailed examination of your lower gastrointestinal tract, commonly referred to as a "2-week wait" or "2ww lower GI" procedure.

We discussed the possibility of cancer and I completely understand your concerns. Rest assured, I will personally call you as soon as the results come in to discuss them with you.

I truly understand that this can be an anxious time, especially with the uncertainty surrounding the symptoms you're experiencing and your family's history. Please remember that we're taking all the necessary steps to get a clear understanding of what's happening. I'm here to support you every step of the way.

Should you have any questions or concerns, please don't hesitate to reach out.

Warm Regards,

[Doctor's Name]

[Doctor's Credentials & Contact Information]

|  |
| --- |

**Case 2: Cancer**

ChatGPT 3.5 Note Nov 1^st^, 2023

|  |
| --- |

Dear [Patient's Name],

I hope this message finds you well. I wanted to reach out to you regarding the recent concerns that you've had about your health. I understand that you've been experiencing episodes of blood mixed with stool over the past few weeks, and I appreciate your courage in seeking medical attention for this issue.

I've reviewed your medical records and the attached photo, and I want to assure you that we are taking your concerns seriously. Your well-being is our top priority. I also noted that you mentioned a change in your body weight, a notch down on your belt buckle, and looser-fitting clothes. While there's no abdominal pain, you've experienced looser bowel movements recently. I'm here to help you address these concerns.

I understand that you have some family history related to your father's passing, but it wasn't discussed in detail, so you're unsure about the specific type of cancer. I want to reassure you that we will investigate your symptoms thoroughly to provide you with the most accurate diagnosis and treatment plan.

During our examination, I didn't find any masses in your abdomen, and there were no masses or signs of blood during the rectal examination. Your current weight is 82kg, and we will continue to monitor this as part of your overall assessment.

Based on your symptoms and medical history, I believe there are two potential scenarios we need to consider: colorectal cancer (CRC) or inflammatory bowel disease (IBD). To help us determine the cause of your symptoms, I have ordered an urgent fecal immunochemical test (FIT). You will find a FIT kit provided to you for this purpose. Please follow the instructions carefully, and we will await the results.

Additionally, I've requested a set of blood tests (U&Es), which you can conveniently get done at the drive-through this afternoon. These tests will provide us with valuable information about your overall health.

Depending on the results of the FIT and the blood tests, it may be necessary for you to undergo a "two-week wait" lower gastrointestinal evaluation. This is a precautionary step to ensure that any potential issues are identified and addressed promptly.

I understand that the possibility of colorectal cancer can be concerning, and I want you to know that I am here to support you throughout this process. Once we have the results of the tests, I will personally reach out to you to discuss the findings and outline the next steps.

Please don't hesitate to reach out if you have any questions or concerns in the meantime. Your health and well-being are of utmost importance to us, and we are committed to providing you with the best possible care.

Take care, and we will be in touch soon.

Sincerely,

[Your Name] [Your Title] [Medical Facility Name]

|  |
| --- |

**Case 2: Cancer**

ChatGPT 4.0 Note Nov 1^st^, 2023

|  |
| --- |

Subject: Clinical Note and Next Steps

Dear [Patient's Name],

I want to thank you for coming in today and discussing your health concerns with me. I understand that this may be a challenging time for you, especially given the symptoms you have been experiencing recently.

Based on our conversation and the examination, it seems that you have been dealing with blood mixed with stool and changes in your bowel habits over the last few weeks. I also noted that you've experienced some unintentional weight loss, as indicated by your clothing fitting more loosely and a change in your belt notch. It is clear that these symptoms have been concerning for you, and I want to assure you that we are taking them seriously.

I am aware that your father passed away from cancer in his 60s, although the specific type of cancer is not clear. I understand that this family history might be adding to your concern at this time. I want to reassure you that we are doing everything we can to get to the bottom of your symptoms and provide you with the care you need.

Based on the examination today, your abdomen was soft, non-tender, and no masses were felt. The rectal examination also did not reveal any masses, and there was no blood on the glove. Your current weight was recorded as 82kg.

In terms of the next steps, I have ordered an urgent Fecal Immunochemical Test (FIT) to check for the presence of blood in your stool that might not be visible to the eye. I have provided you with a pot for the sample, and I would appreciate it if you could return this as soon as possible. Additionally, I have requested a set of blood tests (U&Es) to assess your overall health, which you will be going to the drive-through clinic to complete this afternoon.

I understand that the possibility of cancer, specifically colorectal cancer, is a significant concern. I want to be transparent and honest with you; it is one of the conditions we are considering, given your symptoms. However, it is essential to remember that other conditions, such as Inflammatory Bowel Disease (IBD), could also explain your symptoms. Please know that we are doing everything we can to rule out or confirm these conditions as quickly as possible.

I know this is a lot to take in, and it is completely normal to feel concerned or anxious. I am here for you, and I will call you as soon as the results from the tests are available. Please feel free to reach out if you have any questions or need further clarification on anything we discussed today. Your health and well-being are our top priority, and we are committed to providing you with the best care possible.

Wishing you strength and peace during this time,

[Your Name] [Your Title]

|  |
| --- |

**Case 3: Depression**

Original Note

|  |
| --- |

● History

Seen with mum. Didn’t come home from work on Friday evening. Police brought home at 1am had been on railway bridge. 3 missed calls next morning withheld number (?crisis team). Dropped out of college last year, now working Burger King Meadowhall. Friends have all gone to uni. Things OK at home, lives with mum and younger brother (aged 15). Gets on well with brother. Good relationship with mum. Mum notes more withdrawn recently. Dad moved out >1y. No Hx DV just drifted apart. Not seen dad since new partner 6m ago. Prior to this didn’t talk much, freq away from home with work. Prev studying psychology at college but struggled, missed deadlines. Mum notes pretending to go for a few weeks before letter from college re: non-attendance. Seen college counsellor found it useful. Doesn’t enjoy work, “manager has it in for me”. Works 2-10pm shift. Gets on OK with some work mates but not much in common. Used to play netball but not since leaving college. No hobbies now. Watches TV. Gained weight since starting work lost self-confidence. Was in rel for 2y but bfriend went to uni and lost touch. No recent drug use. Tried a joint once but didn’t like it. Doesn’t drink alcohol. States nothing to look forward to. Mum booked a holiday in Spain Sally trying to get the time off work. Feels numb. Wasn’t seriously considering jumping off bridge, just didn’t want to go home. fleeting thoughts of not wanting to be here, but no specific plans. No Hx self-harm. Feels bad mum and brother were worried about her. Mum keen on antidepressant, Sally not keen

● Examination

Well kempt, quietly spoken, low mood, no evidence of disordered thought, no evidence of hallucinations/delusions, good insight

● Diagnosis

Low mood [uncoded for now]

● Intervention Plan

Discussed options. Would like further counselling. Advised long waiting list. Texted link to IAPT website & crisis numbers. self-help e-mailed to xyzsally@hotmail.com Going to think about joining local netball team but might struggle with hours. Is going to contact Door 43, doesn’t work Tuesdays so could make wellbeing café. See again 2w.

|  |
| --- |

**Case 3: Depression**

ChatGPT 3.5 Note Oct 1^st^, 2023

|  |
| --- |

[Your Name]

[Your Title/Position]

[Date]

Dear Sally,

I hope this message finds you well. I wanted to take a moment to summarize our recent conversation and outline the plan we discussed during your visit to our clinic.

First and foremost, I want to acknowledge your courage in seeking help and sharing your thoughts and feelings with us. It's evident that you've been going through a challenging time, and I want you to know that we are here to support you every step of the way.

During our session, you shared some significant events and feelings that have been affecting you lately. Your experience on the railway bridge was undoubtedly a distressing moment, and I'm glad you're safe. It's clear that you've been under a lot of stress, especially with the recent changes in your life, such as leaving college, adjusting to a new job at Burger King, and your friends moving away to university.

Your relationship with your mother and younger brother is important to you, and it's evident that you care deeply for them. Your mother has noticed that you've become more withdrawn recently, and we discussed the challenges your family has faced since your father moved out. It's understandable that these changes have had an impact on you, and I appreciate your willingness to share your feelings about them.

Your academic struggles and the college counselor's assistance were also discussed. It's essential to recognize that seeking help and utilizing resources like counseling are positive steps toward managing difficulties. Your current work situation, where you feel your manager has been unfair to you, is understandably adding to your stress and affecting your self-confidence.

We also talked about your social life, hobbies, and the loss of your relationship with your previous boyfriend. It's clear that you've been feeling isolated and have lost interest in things you once enjoyed. These feelings of numbness and hopelessness are undoubtedly distressing.

I'm pleased to see that you've been open to discussing your thoughts and feelings with us. Your honesty is a crucial step towards finding support and solutions. After our discussion, we discussed an intervention plan. Here are the key points we discussed:

1. **Counseling**: You expressed interest in further counseling, and I understand that the long waiting list can be discouraging. I've provided you with a link to the IAPT website, which may have additional resources and options for counseling. Please explore this resource, and if you have any questions or need further assistance, don't hesitate to reach out.
2. **Self-Help Resources**: I've also emailed you some self-help materials that may offer guidance during difficult moments. These resources can provide strategies for managing your mood and stress.
3. **Social Engagement**: You mentioned considering joining a local netball team, which is an excellent idea to reconnect with your interests and potentially make new friends. I encourage you to explore this possibility, even if you may face some scheduling challenges.
4. **Door 43 and Wellbeing Café**: You plan to contact Door 43, which is a positive step. Additionally, you noted that you don't work on Tuesdays, which aligns with the Wellbeing Café's schedule. Attending the Wellbeing Café can provide you with a supportive community and additional resources to help you on your journey to improved mental health.

I'd like to see you again in two weeks to discuss your progress and continue working on your well-being. In the meantime, please remember that you are not alone in this journey. If you ever experience an urgent crisis or need someone to talk to, don't hesitate to reach out to the crisis numbers I provided you. Your health and safety are our primary concerns.

I understand that the idea of taking antidepressant medication is not appealing to you at the moment, and that's entirely okay. It's a decision we can revisit and discuss further in the future, if needed, and if you feel more comfortable with the idea.

Sally, please know that we are here to support you, and there is hope for a brighter future. You are a strong and resilient individual, and with the right support, you can overcome these challenges. Don't hesitate to contact us if you have any questions or if you simply need someone to talk to.

Wishing you strength and peace on your journey to recovery.

Warm regards,

[Your Name]

[Your Title/Position]

[Contact Information]

|  |
| --- |

**Case 3: Depression**

ChatGPT 4.0 Note Oct 1^st^, 2023

|  |
| --- |

**Clinical Note for Patient: Sally**

**History**:

Sally, a young woman in her early twenties, presented with her mother. Sally has been experiencing challenges lately that have raised concerns. Recently, Sally had an episode where she did not return home from her work at Burger King in Meadowhall on a Friday evening. She was found by the police on a railway bridge around 1 am. Though she was not seriously considering harming herself, she has acknowledged fleeting thoughts of not wanting to be around.

She dropped out of college last year where she was studying psychology but faced academic challenges, missing deadlines. Sally's mother noted that she had been pretending to go to college even after discontinuing her studies. This led to a consultation with a college counselor, which Sally found beneficial.

She has seen changes in her social environment as her friends have moved on to universities. Furthermore, she's had challenges at work with her manager and doesn't particularly enjoy her job. Though she has some colleagues she gets along with, she feels they don't share many common interests.

Sally's home life has been relatively stable. She lives with her mother and younger brother, aged 15, with whom she has good relationships. However, her father moved out more than a year ago, and they have drifted apart especially since he started a new relationship six months ago.

Personal changes have also affected Sally's well-being. Since starting her job, she has gained weight, leading to a decrease in her self-confidence. She used to be active, playing netball during her college days, but has since discontinued the sport and doesn't engage in any other hobbies now.

Sally's mother suggested the possibility of antidepressants, though Sally is not currently interested in this treatment option.

**Examination**:

Sally appeared well-groomed and spoke in a soft tone. She exhibited signs of low mood. Importantly, there was no evidence of disordered thoughts, hallucinations, or delusions, and Sally demonstrated good insight into her situation.

**Diagnosis**:

Low mood (currently uncoded).

**Intervention Plan:**

Multiple interventions were discussed. Sally expressed an interest in counseling, and resources were provided for her, including a link to the IAPT website and crisis numbers. Suggestions for self-help were emailed to her. Considering her previous interest in netball, she is contemplating joining a local team, though her working hours might pose a challenge. Sally plans to contact Door 43 for the wellbeing café, as she is available on Tuesdays. A follow-up appointment has been scheduled in two weeks.

**Note to Sally:**

Sally, it's evident that you've been through quite a lot in the past year, and it's commendable that you've shown resilience in these trying times. Remember, it's okay to seek help when needed. Everyone's journey is different, and it's essential to find what works best for you. It's promising that you're considering avenues like Door 43 and possibly returning to netball, which could be beneficial both for your well-being and social connections. Looking forward to seeing you in two weeks, and in the meantime, do reach out if needed. Take care.

|  |
| --- |

**Case 3: Depression**

ChatGPT 3.5 Note Nov 1^st^, 2023

|  |
| --- |

[Your Name] [Your Title/Position] [Medical Practice/Hospital Name] [Date]

Dear Sally,

I hope this note finds you in good health and spirits. I wanted to take a moment to discuss our recent meeting and outline our plan moving forward.

Firstly, I want to acknowledge that you have been through a challenging time recently, and I appreciate your willingness to share your thoughts and feelings during our conversation. It's clear that you care deeply for your family and that you've been going through a period of uncertainty and change.

You mentioned your experience on the railway bridge, and I'm relieved to hear that you didn't want to harm yourself but were just struggling with feelings of not wanting to go home. It's completely normal to have moments of despair, especially during difficult times like these. Please remember that you don't have to go through this alone, and there are people who care about your well-being, including your mum and brother.

Your history reveals that you've faced some significant changes in your life, from leaving college to your dad moving out. It's completely understandable that these events have had an impact on your mood and overall well-being. It's positive to hear that you have a good relationship with your family, and your mum's concern for you is a testament to the love and support she has for you.

I'm pleased to see that you've shown an interest in seeking help, and I've provided you with some resources, including the IAPT website and crisis numbers, to assist you in finding the support you need. I've also sent you self-help materials via email, which might offer some guidance during this challenging time.

Your plan to think about joining a local netball team is a positive step. Engaging in physical activity and socializing with others can often help improve one's mood and overall well-being. I understand that your work schedule may make it challenging, but it's worth considering.

Additionally, reaching out to Door 43 and potentially attending the wellbeing café on your free day, Tuesday, is another positive step towards connecting with a supportive community.

We've discussed the option of counseling, and I understand that you have concerns about the waiting list. Please remember that you are not alone in this, and we can explore other potential avenues for support as well. Your well-being is a priority, and I am here to support you throughout this journey.

I want to reiterate that your feelings are valid, and it's okay to seek help when you're going through a tough time. Please reach out to me or any of the provided resources if you ever feel overwhelmed or need someone to talk to. We will have another appointment in two weeks to check in on your progress and discuss any further steps.

Remember, there is hope, and things can get better. You have strengths and resilience within you, and with the right support, you can navigate through these challenging times. Please take care of yourself, Sally, and know that you are not alone on this journey to better mental health.

Sincerely,

[Your Name] [Your Title/Position] [Contact Information]

|  |
| --- |

**Case 3: Depression**

ChatGPT 4.0 Note Nov 1^st^, 2023

|  |
| --- |

Subject: Clinical Summary and Next Steps

Dear Sally,

I wanted to take a moment to summarize our recent appointment and ensure that we are on the same page moving forward. It was great to meet with you and your mum, and I appreciate the openness and honesty you brought to our conversation.

From our discussion, it is clear that you have been facing some challenging times recently. Your transition from college to working at Burger King, coupled with your friends moving away for university, seems to have created a sense of isolation and disconnection. Your mum has also noticed that you have become more withdrawn, and it was concerning for both her and your younger brother when you didn’t come home as expected the other night.

I understand that work has been a source of stress, and you do not feel a strong connection with your colleagues. Additionally, the change in your activity level and interests, such as stopping netball, has also contributed to your current state of mind. It’s clear that these factors, along with the change in your relationship with your dad since he moved out and started a new relationship, have culminated in a period of low mood for you.

It was good to hear that you had a positive experience with a college counsellor in the past, and I’m glad to know that you are open to seeking further counselling. I understand your frustration with the potential wait for these services, but I encourage you to use the resources provided, including the IAPT website and crisis numbers, in the meantime.

I also want to commend you for considering re-engaging in activities that previously brought you joy, such as netball, and for planning to contact Door 43 for additional support. These are positive steps towards regaining a sense of connection and purpose.

Regarding your mum’s suggestion of considering antidepressant medication, it’s important that this decision is made collaboratively and that you feel comfortable with any plan we put in place. We can continue to discuss this option at our next appointment or sooner if you wish.

I want to reassure you that it’s okay to feel the way you are feeling right now, and it’s a sign of strength that you reached out for support. Please remember that you don’t have to navigate this alone; we are here to help and support you through this time.

We have scheduled a follow-up appointment in two weeks, but please do not hesitate to reach out before then if you need to. Your well-being is our top priority.

Take care, and I look forward to seeing your progress in the coming weeks.

Warm regards,

[Your Name] [Your Title and Affiliation]

|  |
| --- |
