# Supplementary material for: Generative artificial intelligence writing open notes: A mixed methods assessment of the functionality of GPT 3.5 and GPT 4.0
Source: Digit Health. 2024 Oct 29;10:20552076241291384. doi: 10.1177/20552076241291384 (PMC11528788; doi:10.1177/20552076241291384)
Supplement: sj-docx-2-dhj-10.1177_20552076241291384 - Supplemental material for Generative artificial intelligence writing open notes: A mixed methods assessment of the functionality of GPT 3.5 and GPT 4.0 [file sj-docx-2-dhj-10.1177_20552076241291384.docx]

**Appendix 2. Descriptive metrics of October 2023 and November 2023 GPT Notes**

## **Linguistic metrics**

**Table 1**. Linguistic metrics of GPT notes from October 1, 2023 and November 1, 2023.

|  | **ChatGPT 3.5 Oct ‘23** | | | **ChatGPT 3.5 Nov ‘23** | | | **ChatGPT 4.0 Oct ‘23** | | | **ChatGPT 4.0 Nov ‘23** | | |
| --- | --- | --- | --- | --- | --- | --- | --- | --- | --- | --- | --- | --- |
|  | Diabetes | Depression | Cancer | Diabetes | Depression | Cancer | Diabetes | Depression | Cancer | Diabetes | Depression | Cancer |
| **Total words**, *n* | 488 | 721 | 479 | 496 | 522 | 465 | 373 | 506 | 355 | 383 | 450 | 479 |
| **WPS**, *n* | 18.77 | 18.02 | 19.16 | 19.08 | 20.08 | 17.88 | 15.54 | 14.88 | 16.9 | 18.24 | 22.5 | 20.83 |
| **Pronouns**, *%* |  |  |  |  |  |  |  |  |  |  |  |  |
| 1^st^ person, sg. | 3.07 | 1.94 | 1.67 | 2.62 | 2.49 | 3.87 | 1.34 | – | 2.25 | 3.13 | 2 | 3.55 |
| 1^st^ person, pl. | 2.87 | 2.22 | 2.71 | 3.23 | 1.15 | 2.8 | 2.95 | – | 1.13 | 2.35 | 2.22 | 2.09 |
| 2^nd^ person | 6.97 | 10.4 | 8.77 | 7.06 | 9.39 | 8.6 | 4.83 | 1.19 | 8.45 | 6.53 | 8 | 7.52 |
| 3^rd^ person, sg. | – | – | 0.21 | – | 0.19 | – | – | 7.71 | 0.28 | – | 0.44 | 0.21 |
| 3^rd^ person, pl. | – | 0.28 | 0.21 | – | – | – | – | 0.4 | 0.28 | – | – | 0.21 |
| **Abbreviations**, *n* | 3 | 1 | 5 | 4 | 1 | 4 | 11 | 1 | 8 | 4 | 1 | 3 |
| De-abbreviations | 1 | – | 5 | 2 | – | 4 | 8 | – | 7 | 2 | – | 3 |

## Note: WPS – words per sentence. Pronouns detected by LIWC-22: 1^st^ person, sg. – I, me, my, myself; 1^st^ person, pl. – we, our, us, lets; 2^nd^ person – you, your, u, yourself; 3^rd^ person, sg. – he, she, her, his; 3^rd^ person, pl. – they, their, them, themsel*. In the count of abbreviations, only unique instances were included and ‘XY’ was excluded as it was placeholder initials for the fictitious note.

## **Readability metrics**

**Table 2**. Readability metrics of GPT notes from October 1, 2023 and November 1, 2023.

|  | **ChatGPT 3.5 Oct ‘23** | | | **ChatGPT 3.5 Nov ‘23** | | | **ChatGPT 4.0 Oct ‘23** | | | **ChatGPT 4.0 Nov ‘23** | | |
| --- | --- | --- | --- | --- | --- | --- | --- | --- | --- | --- | --- | --- |
|  | Diabetes | Depression | Cancer | Diabetes | Depression | Cancer | Diabetes | Depression | Cancer | Diabetes | Depression | Cancer |
| **Flesch Reading-Ease** | | | | | | | | | | | | |
| Score | 55.2 | 57.3 | 51.9 | 56.9 | 59.6 | 57.6 | 63 | 56.2 | 54.6 | 61.1 | 58 | 61.8 |
| US grade level | 10^th^ - 12^th^ | 10^th^ - 12^th^ | 10^th^ - 12^th^ | 10^th^ - 12^th^ | 10^th^ - 12^th^ | 10^th^ - 12^th^ | 8^th^ - 9^th^ | 10^th^ - 12^th^ | 10^th^ - 12^th^ | 8^th^ - 9^th^ | 10^th^ - 12^th^ | 8^th^ - 9^th^ |
| **Flesch-Kincaid Grade Level** | | | | | | | | | | | | |
| Score | 10.1 | 9.8 | 10.8 | 10 | 9.9 | 9.7 | 7.8 | 9.2 | 9.9 | 9 | 10.8 | 9.8 |
| **Gunning Fox Index** | | | | | | | | | | | | |
| Score | 13.3 | 12.5 | 10.8 | 13 | 12.9 | 12.9 | 9.2 | 9.2 | 9.9 | 11.9 | 14.3 | 12.7 |
| US grade level | College freshman | High-school senior | College sophomore | College freshman | High-school senior | High-school senior | High-school freshman | High-school senior | High-school senior | High-school senior | College freshman | College freshman |

## Note: For the Flesch Reading-Ease test, the higher the score the easier the text is to read. For the Flesch-Kincaid Grade Level, the score represents a US grade level. For the Gunning Fox Index, the higher the score the more difficult the text is to read.

## **Medical fidelity**

**Table 3**. Medical fidelity measures of GPT notes from October 1, 2023 and November 1, 2023.

|  | **ChatGPT 3.5 Oct ‘23** | | | **ChatGPT 3.5 Nov ‘23** | | | **ChatGPT 4.0 Oct ‘23** | | | **ChatGPT 4.0 Nov ‘23** | | |
| --- | --- | --- | --- | --- | --- | --- | --- | --- | --- | --- | --- | --- |
|  | Diabetes | Depression | Cancer | Diabetes | Depression | Cancer | Diabetes | Depression | Cancer | Diabetes | Depression | Cancer |
| **Would you use the note generated by ChatGPT unchanged as it is?** *n (%)* | | | | | | | | | | | | |
| Yes | 0 | 2 (66.6) | 5 (100) | 1 (16.6) | 0 | 0 | 3 (50) | 6 (100) | 6 (100) | 1 (16.6) | 0 | 1 |
| No | 6 (100) | 4 (33.3) | 0 | 5 (83.3) | 6 (100) | 6 (100) | 3 (50) | 0 | 0 | 5 (83.3) | 6 (100) | 5 (83.3) |
| **How well does ChatGPT preserve the clinical detail of the original note?***, median* | | | | | | | | | | | | |
| Rating | 5 | 6 | 7 | 6 | 5 | 6.5 | 7 | 6 | 6 | 6 | 5 | 6.5 |

## Note: The Likert scale to rate the preservation of clinical detail ranged from ‘1 – Not at all’ to ‘7 – Fully preserves clinical detail’. Calculations were made excluding missing data.
